# The impact of social capital on health behaviors: evidence from urban China

**DOI:** 10.3389/fpubh.2025.1525075

**Published:** 2025-06-24

**Authors:** Long Niu, Chuntian Lu, Xiaojuan Quan

**Affiliations:** ^1^Center for Physical Education, Xi’an Jiaotong University, Xi’an, China; ^2^Department of Sociology, School of Humanities and Social Sciences, Xi’an Jiaotong University, Xi’an, China; ^3^School of Marxism, Xi’an Jiaotong University, Xi’an, China

**Keywords:** social capital, health behaviors, physical exercise, dietary socialization, positive and negative effects

## Abstract

**Background:**

Social capital plays a crucial role in sustaining individual health behaviors. While numerous studies have confirmed the positive impact of social capital on individual health outcomes, limited research has explored its influence on specific health behaviors. Based on data from Job Search and Social Networks 2014 (JSNET 2014) in eight China cities, this study assesses social capital through the occupational heterogeneity of the Bainian Network and the breadth of participation in the Dining Network, aiming to analyze the influence of social capital on health behaviors.

**Methods:**

First, given that the dependent variable is ordinal, this study employs an ordinal logistic regression model (ordered logistic model) to estimate the cumulative odds of social capital affecting health behaviors. Second, it examines the relationship between social capital and positive health behaviors, including exercise frequency and routine health checkups. Finally, it explores the association between social capital and negative health behaviors, specifically the frequency of alcohol consumption and smoking. All data analyses were conducted using Stata 17.0.

**Results:**

(1) Social capital significantly influences both positive and negative health behaviors. (2) There is a positive correlation between social capital and health-promoting behaviors, such as physical activity and routine medical checkups. (3) The relationship between social capital and negative health behaviors-such as increased alcohol consumption and smoking frequency-underscores the dual nature of social networks, which can either foster positive behaviors or reinforce unhealthy habits.

**Conclusion:**

The findings suggest that social capital has a dual impact: it promotes positive health behaviors such as physical exercise and regular health checkups, while also reinforcing negative health behaviors like drinking and smoking. Nonetheless, these findings are limited to urban population, and future research should extend to rural population to provide a more comprehensive understanding.

## Introduction

Health is fundamental prerequisite for human well-being. To improve health and promote health equity, it is insufficient to focus solely on the biological causes of disease. Evidence from chronic disease and epidemiological research indicates that the primary determinants of human health are deeply embedded in the institutional and structural dimensions of social life (1). The World Health Organization’s Commission on the Social Determinants of Health asserts that the social conditions in which people live and work are fundamental causes of disease. This has led to the development of a comprehensive conceptual framework known as the ‘Social Determinants of Health.’ Within this framework, improving daily living environments and addressing social structural factors have been identified as two primary strategies for action (2). Social capital, as a key concept reflecting human connectivity, effectively captures critical aspects of both daily life and the broader social structure. Consequently, whether and how social capital influences health has become as a siginificant academic concern, with research in this area growing rapidly.

The relationship between social capital and health has been extensively discussed and investigated in academic research. Numerous studies have shown that there is a positive correlation between social capital and health- that is, groups with higher levels of social capital tend to exhibit better average health outcomes compared to those with lower levels. This supports a widely accepted perspective known as the “positive facilitation theory,” which suggests that social capital promotes health across multiple dimensions. Specifically, social capital has been shown to reduce mortality through social integration and social trust (3). Additionally, it serves as a social resource that helps mitigate adverse mental conditions, thereby enhancing self-rated health (4). However, disparities in social capital stocks contribute to differences in people’s health status. Although previous studies have shown that social capital has a significant positive impact on health, most of them have focused on the direct impact of social capital on health outcomes - such as mortality, morbidity, and self-rated health - while paying insufficient attention to health behaviors (5, 6). This makes it difficult to explain how behavioral choices can become a “black box” for the transformation of social capital into health outcomes. Furthermore, the measurement dimensions of social capital used in many studies are mostly inherited from Western theoretical frameworks (e.g., Putnam’s Community Participation Index, Coleman’s Trust and Norms Scale) (7, 8). These approaches often lack targeted consideration of social interaction patterns with Chinese characteristics-such as dietary socialization, humanistic networks, and familial ties (9). As a result, their theoretical explanatory power is limited in cross-cultural contexts.

In China, increasing attention is being paid to people’s health and well-being. Proactive disease prevention and public health interventions have emerged as key strategic choices to improving people’s health, particularly in the context of an undeveloped healthcare system and limited medical resources. Therefore, Healthy China Action 2019–2030 emphasizes the need to “move the gate forward” by prioritizing prevention and striving to keep people from disease. Among various social factors, some scholars have noticed that the health effect of social capital in the early 2000s (10), leading to the emergence of several high-quality studies. However, most of these studies have focused on self-rated physical or mental health outcomes, rather than on health behaviors themselves. Meanwhile, in terms of the measurement of social capital, existing studies have mainly focused on the effects of generalized social capital - such as network structure, relationship resources, and bond strength - while pay insufficient attention to culturally specific forms of social capital in the Chinese context, such as dietary socialization. Addressing this gap, this study examines the impact of social capital on both positive health behaviors, such as physical exercise and health checkups, and negative health behaviors, including drinking and smoking. By incorporating culturally relevant dimensions of social capital, this study aims to provide empirical evidence and policy implications for public health strategies in developing countries like China.

## Literature review and research hypotheses

### Research in social capital

Social capital is a broad concept, that refers both to norms such as trust and participation at the collective level (11, 12), and to resources embedded in individual social networks (13, 14). While the former influences actors and their behaviors at a broader social scale like neighborhood, community, region, etc., the latter mainly emphasizes the flow and function of resources within individual social networks. In the Chinese contect, interpersonal relationships are often regarded as the key to understanding social structure and culture. For example, Liang positioned Chinese culture and society as “ethical” (15), where love and righteousness are the main focus, while Fei described the Chinese society as a “differential mode of association,” distinguishing it from West models (16). The main meaning of “face,” which widely exists in popular discourse and academic research, is the maintenance and accumulation of social relations. Based on this, Bian proposed the concepts of “relational sociology” and “relational social capital” within the framework of comparing the East and the West and the internationalization of Chinese sociology. These concepts highlight the behavioral meanings and mechanisms of social capital embedded in China’s relational culture (17, 18). In this study, we adopt the concept of social capital at the individual level in order to examine its influence on health-rated behaviors.

Zhao (19) provided a detailed review of individual social capital measurement, which will not be repeated it here. Instead, we briefly explain the measurement methods in the Chinese context. Two main methods are commonly used to measure individual social capital, that is, the structure of an individual’s social network: name generator and position generator. The network measured by name generator vary according to the question direction, e.g., a person’s “job-seeking network” “lending network” and “discussion network” are networks composed of different relationships. Moreover, this method typically limits the respondent’s network to 3–5 individuals, which underrepresents the larger and more complex social capital and network. To address this limitation, Bian proposed position generator based on Spring Festival visitation practices-an important cultural ritual of paying New Year’s visits - and termed the resulting network “Bainian network” (20). Related studies have shown that the Bainian network is a powerful indicator of social capital in the Chinese context, with greater validity than the discussion network (21). In fact, this measurement was introduced into the 2003 China General Social Survey (CGSS) as a culturally specific indicator of Chinese social relations.

In addition, because Chinese reciprocal interactions are often realized through gathering and dining, the “dining network” has also been used as an operationalized concept of social capital in the Chinese context (22–24). This network captures dimensions of social resources that differ from those reflected in the “Bainian network” (25). There have been numerous research has explored the characteristics and functions of dining networks (26, 27). It should be noted that dining networks are social in nature, including three modes of interaction, inviting others, being invited, and accompanying - distinct from general notions of dining ou. In this paper, we conceptualize the dining network as an important process and way of building, maintaining and mobilizing social capital in the Chinese context (28).

### Social capital and health

The core connotation of social capital lies in interpersonal connectedness, and its impact on health can be traced back to Durkheim’s discussion related to social solidarity and suicide (29). And since Howes et al. (1988) explicitly identified social relationships as a significant determinant of health in Science, numerous studies have been devoted to explaining and validating the existence of this association (30, 31).

However, in examining the relationship between social structures and health, scholars have used different concepts-such as social support, social cohesion, social integration, and social network - with some scholars grouping these terms under the concept of social capital (32). This paper adopts this strategy by measuring social capital from the perspective of social networks, based on two key considerations. On the one hand, social networks represent both the quantity and quality of social support, reflecting the degree of social cohesion and social integration, and affecting people’s health (33). On the other hand, social network may exert independent effects on health, i.e., the social relationships itself (rather than the social support embedded in it) may also affect health (34).

Numerous studies have shown that social capital has a significant impact on mortality, self-rated health, illness risk, and health behaviors (10, 35). While most studies emphasize the health-promoting effects of social networks, there is also substantial evidence indicating that social network may contribute to the adoption of unhealthy behaviors and outcomes. For example, Christaki and Fowler found that obesity is associated with social networks and can spread through social ties (36). Similar findings have been observed in studies on drinking and smoking behaviors (37, 38). In other words, social networks are double-edged in nature, which can both enhance and impair people’s health.

Chinese scholars noticed the health effects of social capital in the early 21st. In particular, its impact has received special attention in studies focusing on specific populations, such as, adolescents, older adults, and migrant groups (39–42). These findings are based on nationally or regionally representative data. For example, Pan’s analysis showed that social capital has a significant effect on both self-rated physical and mental health, although this effect varies across different demographic groups (43). Furthermore, some studies have highlighted the dual nature of social capital, noting both its positive and negative health implications. For instance, Zhou et al. showed that social capital not only has a significant positive effect on population health but also helps mitigate the negative impact of income disparity on health (44).

### Research questions and hypotheses

Overall, existing research has significant value in understanding the health effects of social capital; however, notable limitations remain. For instance, the study of health behaviors, which this paper focuses on and emphasizes, has been relatively underexplored in China, with most research primarily focusing on health outcomes such as self-rated physical or mental health. While such studies help elucidate the broader patterns of social capital, they do not directly capture its influence on health behaviors. A focus on health behaviors, rather than health outcomes alone, would offer greater policy relevance, as it can help identify and mitigate the promotion of unhealthy behaviors by social capital at an earlier stage, ultimately enhancing the positive effects of social capital on health. Moreover, existing studies on social capital and health generally adopt measurement framework developed in Western contexts, paying insufficiently attention to China’s unique sociocultural context and the specific expressions of social capital, such as dietary socialization. Therefore, this paper proposes a research framework aimed at preliminary analyzing the impact of social capital on health behaviors within the Chinese context.

It is well known that physical activity plays a vital role in promoting human health and well-being. Regular physical activity is the key to preventing heart disease, type II diabetes, and cancer. It is also effective in reducing symptoms of depression and anxiety, improving memory, and enhancing brain function. However, only 33.9% of urban and rural residents in China regularly participate in physical activity, making physical inactivity one of the major contributing factors to the rising prevalence of chronic diseases (45). At the same time, excessive alcohol consumption poses significant health risks. China has a long-standing alcohol culture deeply rooted in social and historical traditions (46). Since the Reform and Opening-Up of China, drinking has become a “self-help action” to reduce uncertainty, and has gradually solidified into a “social form” (47). In addition, smoking is not only a leading cause of pulmonary diseases,” but also significantly increases the risk of cardiovascular disease and stroke. One in three smokers die from smoking-related diseases, and on average, smokers have a life expectancy that is 10 years shorter than that of non-smokers (48). Globally, tobacco use causes over 8 million death annually, including 1.2 million deaths among non-smokers due to secondhand smoke exposure (49). Therefore, this paper focused on three critical behaviors: physical activity, alcohol consumption, and smoking. At the same time, health checkups are also included in the analysis, due to their important role in early detection, treatment and health management. In this study, physical activity and health checkups are categorized as positive health behaviors, while alcohol consumption and smoking are classified as negative health behaviors.

This paper posits that social capital significantly influences individuals’ health behaviors. Variations in the structural resources of social capital, as well as differences in the processes of maintaining, consolidating, strengthening, and expanding these resources, can lead to disparities in health behaviors.

On the one hand, social capital provides access to supportive relationships, health-related knowledge, and information, thereby promoting positive health behaviors (50, 51). For example, Zhao and Hu found that a higher proportion of strong ties within the social networks of new mothers increased the likelihood of receiving practical support, which in turn enhanced the probability of breastfeeding. Moreover, connections with medical professionals facilitated more effective knowledge transmission, further improving breastfeeding outcomes (52). At the same time, social capital may also facilitate the diffusion of negative health behaviors. This is because social capital encompasses not only supportive norms and resources but also behaviors, attitudes, and values that may be harmful to health. The very connectedness that enables the dissemination of positive health practices can similarly amplify the spread of detrimental ones. Therefore, the following hypothesis is proposed:

*Hypothesis 1.1*: The structural resources of social capital have a significant impact on health behaviors. The richer the structural resources of social capital, the greater the prevalence of positive health behaviors among individuals.

*Hypothesis 1.2*: The richer the structural resources of social capital, the greater the prevalence of negative health behaviors among individuals.

On the other hand, the process of maintaining, consolidating, reinforcing, and broadening social capital - such as dietary socialization - also exert important influences on health behaviors. Dietary socialization performs both affective and instrumental functions (53). These functions include the dissemination of health-related information, which may be made more effective by the presence of “shared emotions” (54, 55), both positive and negative. Moreover, one of the important purposes of food socialization is goal achievement, i.e., to express “goodwill” through feasting and participation in feasting, with the aim of “establishing or bringing relationships closer” or even “making exchanges” (56). In this process, imitation mechanisms and social norms mechanisms will work together to influence people’s behaviors, such as drinking and smoking. Therefore, the following hypothesis is proposed:

*Hypothesis 2.1*: The processes of maintaining, consolidating, strengthening, and expanding social capital have a significant impact on health behaviors. The greater the extent of maintaining, consolidating, strengthening, and expanding social capital, the more positive health behaviors individuals will exhibit.

*Hypothesis 2.2*: The greater the extent of maintaining, consolidating, strengthening, and expanding social capital, the more negative health behaviors individuals will exhibit.

## Data and variable measurement

### Data sources

To test the proposed hypotheses, this study draws upon data from the “Job Search and Social Networks 2014” (JSNET 2014), conducted by the Institute of Empirical Social Science at Xi’an Jiaotong University. The survey included both permanent and temporary residents from urban areas in Changchun, Lanzhou, Xi’an, Tianjin, Jinan, Shanghai, Xiamen, and Guangzhou. Utilizing a cross-sectional design, the study employed a multi-stage stratified Probability Proportional to Size (PPS) sampling method to ensure representative sampling. Data collection was implemented via computer-assisted personal interviews, yielding a valid sample of 5,480 respondents. Although JSNET 2014 does not constitute a longitudinal panel, include a tracking panel, its sampling framework was rigorously constructed using the 6th Population Census Matrix from the National Bureau of Statistics of China (NBSC). Post-stratification was applied to ensure alignment with national demographic benchmarks in terms of age, education (ranging from illiteracy to PhD), and occupational sector. At present, the JSNET 2014 dataset is widely regarded as one of the most influential, widely utilized, and authoritative sources of social capital survey data.

### Variable measurement

#### Dependent variables

Positive health behaviors and negative health behaviors constitute the two main categories of dependent variables in this study. Positive health behaviors include two variables: frequency of health check-ups and exercise frequency. Negative health behaviors include two variables: frequency of alcohol consumption and smoking frequency.

To measure health check-up behavior, The JSNET 2014 survey asked: “In the past 3 years, have you undergone any health check-ups? “with response options: 1 = Yes, regularly; 2 = Yes, but not regularly; 3 = No check-ups. To facilitate interpretation, responses were reverse-coded to generate an ordinal variable, with higher values indicating more frequent and regular participating in health check-ups. Exercise frequency was assessed by asking how often respondents engaged in physical activity lasting at least 20 min. Alcohol consumption was measured by the reported frequency of drinking. Both questions used the following response categories: 1 = Daily; 2 = Several times a week; 3 = Several times a month; 4 = Several times a year or less; 5 = Never. Smoking frequency was assessed with the item: “How often do you smoke?” Responses included: 1 = A pack or more daily; 2 = About one pack every 2 days; 3 = 1–2 packs a week; 4 = Occasionally; 5 = Never. For all three behaviors - exercise, alcohol consumption, and smoking - reverse coding was employed to generate three ordinal variables, where higher values represent higher frequencies of each behavior.

#### Independent variables

The occupational heterogeneity of the “New Year Visit Network” and the frequency of dining social interactions are the two core independent variables in this study. Drawing on prior literature, both are recognized as culturally appropriate and context-specific operationalizations of social capital in China. The occupational heterogeneity of the New Year Visit Network is measured by the number of distinct occupational categories among individuals with whom respondents interacted during the Spring Festival visits. A greater number of occupational categories signifies higher structural heterogeneity, reflecting broader social connections and access to more diverse, non-redundant resources. This diversity enhances the quality and utility of social capital. Conversely, the dining network captures a crucial process through which individuals maintain, strengthen, and expand their social relationships, thereby mobilizing social capital. Consequently, a higher frequency of dining social interactions correlates with broader social connections and greater mobilizable social capital. Unlike network differences, the frequency of social dining has a more direct influence on non-healthy behaviors such as drinking and smoking.

The JSNET 2014 survey asked respondents to estimate the number of relatives, friends, and others with whom they interacted during the Spring Festival through various forms of New Year greetings and social engagements. Based on this, the study applied the positioning method to measure the occupational distribution of the respondents’ social network members. This process generated a variable indicating the occupational heterogeneity of the New Year Visit Network, referred to as network diversity. A higher value for this variable indicates greater network diversity and a higher potential quality of social capital (57). Additionally, the questionnaire included three items measuring how frequently respondents hosted others for meals, were invited to meals, and accompanied others to meals (18, 58). Each items used a five-point Likert scale: 1 = Never, 2 = Rarely, 3 = Sometimes, 4 = Often, 5 = Very Often. Although these questions did not directly capture the social characteristics of participants, they effectively reflect the strength and intensity of social network. By summing the scores of the three items, a composite variable representing the frequency of dining social interactions was created, ranging from 0 to 15. Higher scores indicate more frequent participation in such interactions and, consequently, a greater capacity to mobilize social capital.

#### Control variables

Furthermore, to control for potential confounding effects from other social factors and to more precisely estimate the impact of social capital on health behaviors, this study included a range of demographic and socioeconomic variables as control variables. These included gender, age, household registration status, ethnicity, years of education, the logarithm of annual household income, and marital status. Table 1 presents the descriptive statistics for all dependent, independent, and control variables. Given that the frequency of exercise, health check-ups, drinking, and smoking frequency are all ordinal variables, the analysis employed an Ordered Logit Model (also known as the Proportional Odds Model), which estimates cumulative probabilities rather than the probability of selecting a specific category. All statistical analyses were conducted using Stata 17.0.

**Table 1:** Descriptive statistics of variables.

Variables	Mean	Std. Dev.	Categories and value ranges
Dependent variable			
Positive health behaviors			
Exercise	\	\	1 = (Never exercise, 18.54%), 2 = (Once a year or less, 11.06%), 3 = (A few times a month, 17.15%), 4 = (A few times a week, 24.15%), 5 = (Exercise every day, 29.10%)
Health check-ups	\	\	1 = (No health check-ups, 41.36%), 2 = (Irregular health check-ups, 32.88%), 3 = (Regular health check-ups, 25.76%)
Negative health behaviors			
Drinking	\	\	1 = (Never drink alcohol, 59.29%), 2 = (A few times a year or less, 15.43%), 3 = (A few times a month, 13.84%), 4 = (A few times a week, 7.13%), 5 = (Drink every day, 4.31%)
Smoking	\	\	1 = (Never smoke, 73.91%), 2 = (Occasionally smoke, 5.28%), 3 = (1–2 packs a week, 2.67%), 4 = (About one pack every 2 days, 7.04%), 5 = (One pack or more every day, 11.10%)
Independent variable			
Network heterogeneity	4.88	4.169	0 ~ 20
Social dining frequency	7.236	2.979	0 ~ 15
Control variables			
Gender	0.472	0.499	0 = (Female, 52.83%), 1 = (Male, 47.17%)
Age	43.56	13.76	16–84
Household registration	0.876	0.329	0 = (Agricultural household registration, 12.39%), 1 = (Non-agricultural household registration, 87.61%)
Ethnicity	0.967	0.179	0 = (Non-Han ethnicity, 3.32%), 1 = (Han ethnicity, 96.68%)
Years of education	12.812	3.357	0 ~ 19
Annual household income	1.951	0.928	−2.303 ~ 4.784
Marital status	\	\	0 = (Unmarried and divorced, 28.49%), 1 = (Married, 71.51%)

The annual household income has been log-transformed and is measured in units of 10,000 RMB.

## Results analysis and interpretation

Tables 2, 3 present the results of the impact analysis of social capital on positive and negative health behaviors, respectively. For each health behavior, two models are estimated. Model (1) includes only the control variables, while Model (2) extends Model (1) by incorporating the social capital variables. The relevant results are discussed in detail below.

**Table 2:** Regression analysis results of social capital and positive health behaviors.

Variables	Model (1)	Model (2)	Model (3)	Model (4)
	Exercise		Health Check-up	
Gender (Ref: female = 0)	0.165*** (0.049)	0.120** (0.050)	0.054 (0.052)	−0.022 (0.053)
Age	0.039*** (0.002)	0.040*** (0.002)	0.009*** (0.002)	0.011*** (0.002)
Household registration (Ref: Agricultural household registration = 0)	0.090 (0.082)	0.137* (0.082)	0.446*** (0.083)	0.502*** (0.084)
Ethnicity (Ref: Non-Han ethnicity = 0)	−0.056 (0.140)	−0.059 (0.140)	0.112 (0.143)	0.130 (0.144)
Marital Status (Ref: Unmarried and divorced = 0)	−0.269*** (0.058)	−0.306*** (0.059)	0.317*** (0.062)	0.300*** (0.062)
Years of Education	0.045*** (0.009)	0.034*** (0.009)	0.137*** (0.010)	0.121*** (0.010)
Annual household income	0.044 (0.029)	0.017 (0.030)	0.331*** (0.031)	0.279*** (0.032)
Network heterogeneity		0.047*** (0.006)		0.055*** (0.007)
Social dining frequency		0.007 (0.010)		0.039*** (0.010)
Cut point 1	2.072*** (0.219)	2.202*** (0.229)	2.352*** (0.227)	2.714*** (0.238)
Cut point 2	3.160*** (0.222)	3.297*** (0.231)	3.898*** (0.231)	4.283*** (0.242)
Cut point 3	0.672*** (0.218)	0.790*** (0.227)		
Cut point 4	1.302*** (0.218)	1.425*** (0.228)		
Loglikelihood	−8145.71	−8114.86	−5544.23	−5494.34
*N*	5,324	5,324	5,421	5,421

*** *p* < 0.01, ** *p* < 0.05, * *p* < 0.1; Standard errors are shown in parentheses.

The annual household income has been log-transformed and is measured in units of 10,000 RMB.

**Table 3:** Regression analysis results of social capital and negative health behaviors.

Variables	Model (1)	Model (2)	Model (3)	Model (4)
	Drinking		Smoking	
Gender (Ref: female = 0)	2.090*** (0.061)	2.022*** (0.062)	3.458*** (0.109)	3.402*** (0.109)
Age	−0.012*** (0.003)	−0.005* (0.003)	−0.005 (0.003)	0.001 (0.003)
Household registration (Ref: Agricultural household registration = 0)	−0.361*** (0.089)	−0.322*** (0.090)	0.019 (0.112)	0.075 (0.114)
Ethnicity (Ref: Non-Han ethnicity = 0)	0.439*** (0.170)	0.482*** (0.171)	0.237 (0.201)	0.246 (0.200)
Marital status (Ref: Unmarried and divorced = 0)	0.050 (0.069)	0.071 (0.070)	0.275*** (0.087)	0.292*** (0.088)
Years of education	0.010 (0.010)	−0.019* (0.011)	−0.117*** (0.012)	−0.136*** (0.013)
Annual household income	0.148*** (0.033)	0.049 (0.035)	−0.123*** (0.041)	−0.207*** (0.043)
Network heterogeneity		0.043*** (0.007)		0.032*** (0.009)
Social dining frequency		0.135*** (0.011)		0.092*** (0.014)
Cut point 1	1.486*** (0.255)	2.469*** (0.269)	1.880*** (0.307)	2.594*** (0.323)
Cut point 2	2.386*** (0.257)	3.406*** (0.271)	2.291*** (0.307)	3.013*** (0.324)
Cut point 3	3.516*** (0.259)	4.593*** (0.275)	2.513*** (0.308)	3.240*** (0.324)
Cut point 4	4.635** (0.265)	5.742** (0.281)	3.221** (0.308)	3.960** (0.326)
Loglikelihood	−5750.89	−5638.24	−3923.86	−3886.95
*N*	5,423	5,423	5,422	5,422

*** *p* < 0.01, ** *p* < 0.05, * *p* < 0.1; Standard errors are shown in parentheses.

The annual household income has been log-transformed and is measured in units of 10,000 RMB.

### The impact of social capital on positive health behaviors

As shown in Table 2, Model (1) and Model (2) present the analysis results for exercise frequency. Model (1) serves as the baseline model, incorporating only innate control variables such as gender, age, household, years of education, household income, marital status, etc. This model aims to isolate the baseline effects of individual innate attributes on health behaviors (e.g., males may exhibit higher exercise frequency due to physical strength advantage). The results of Model (1) indicates that the coefficients for gender, age, marital status, and years of education are all significant at the 0.01 level, suggesting that these variables exert a substantial influence on exercise behaviors. In the Ordered Logit (Ologit) model, a positive regression coefficient implies that an increase in the independent variable is associated with a higher probability of the dependent variable falling into a higher category, and a lower probability of it falling into a lower category. Conversely, a negative regression coefficient indicates the opposite effect. Specifically, the coefficient for gender is 0.165 (more than 0), indicating that males have a higher frequency of exercise than females. Similarly, as age and years of education increase, individuals’ exercise frequency also tends to rise. In contrast, the coefficient for marital status is −0.269 (less than 0), indicating that married individuals are less likely to exercise less frequently compared to their unmarried or divorced counterparts.

Model (2) adds network differences and social dining frequency to the baseline Model (1), which controls for individual-level antecedent factors. This allows for the assessment of the independent contribution of posteriori social factors to health behaviors by comparing model fit and coefficients stability. The results demonstrate a notable improvement in model performance: the log-likelihood value increases from −8145.71 to −8114.86, indicating a significant enhancement in explanatory power (LRchi^2^ (2) = 61.70, Prob > chi^2^ = 0.000).

This improvement is primarily driven by the contribution of network diversity, which significantly increases individuals’ exercise frequency (*β* = 0.047, *p* < 0.01), thereby supporting Hypothesis 1.1—that richer structural social capital resources are associated with more frequent engagement in positive health behaviors. Although the coefficient for social dining frequency is positive (0.007), it does not reach statistical significance, indicating that Hypothesis 2.1 is not supported.

Additionally, compared to Model (1), the direction and significance of the effects of gender, age, marital status, and education on exercise frequency remains consistent. However, the coefficient for household registration becomes significant (*β* = 0.137, *p* < 0.1), indicating that individuals with non-agricultural household registration are more likely to engage in frequent exercise than those with agricultural registration. This suggests that differences in social capital may obscure the variations in physical activity behaviors among different household registration types.

Models (3) and (4) present the analysis results for health check-up behaviors. Firstly, the effects of age, household registration, and years of education on health check-up behaviors align with their effects on exercise frequency. However, the impact of marital status is reversed in this context. Additionally, gender and ethnicity do not significantly affect health check-up behaviors, indicating no notable differences between males and females or between Han and non-Han individuals in terms of health check-up participation. Moreover, an increase in the logarithm of household income significantly enhances the likelihood of individuals undergoing health check-ups. Secondly, the coefficients for network diversity and social dining frequency are 0.055 and 0.039, respectively, both statistically significant at the 0.01 level. This suggests that increases in network diversity and social dining frequency both enhance the likelihood of individuals participating in health check-ups, including regular check-ups. Further tests indicate that Model (4) fits better than Model (3) (LR chi^2^ (2) = 99.79, Prob > chi^2^ = 0.000). In other words, social capital effectively explains variations in health check-up behaviors and significantly influences these behaviors. Therefore, both Hypothesis 1.1 and Hypothesis 2.1 are supported: richer structural resources of social capital and more extensive processes of maintaining, consolidating, strengthening, and expanding social capital are associated with greater engagement in positive health behaviors.

### The impact of social capital on negative health behaviors

Table 3 presents the analysis results regarding the impact of social capital on negative health behaviors. Models (1) and (2) examine the frequency of alcohol consumption. A comparison of these models reveals that the coefficients for gender and ethnicity consistently remain positive and statistically significant at the 0.01 level. This indicates that males are more likely to consume alcohol more frequently than females, and Han individuals tend to have higher frequencies of alcohol consumption compared to their non-Han counterparts. Additionally, the coefficients for age and household registration status are both significantly negative, suggesting that as age increases, individuals are more likely to belong to lower frequency categories for alcohol consumption. Moreover, individuals with non-agricultural household registration are more likely to fall into lower alcohol consumption frequency categories relative to those with agricultural registration. Furthermore, the effect of educational attainment is not significant in Model (1) but becomes significant in Model (2), indicating that its impact on alcohol consumption frequency is obscured by the differences in social capital. Conversely, the effect of household income (logged) is significant in Model (1) but loses significance in Model (2), suggesting that its influence on alcohol consumption frequency is primarily operates through social capital.

The coefficients for network diversity and social dining frequency are 0.043 and 0.135, respectively, both significant at the 0.01 level, indicating that these factors increase the likelihood of individuals engaging in higher frequencies of alcohol consumption. An increase in network diversity and social dining frequency contributes to a rise in drinking behaviors. Moreover, the Loglikelihood value for Model (2) improves by 112.65 compared to Model (1) (LR chi^2^ (2) = 73.81, Prob > chi^2^ = 0.000), demonstrating a superior fit of Model (2). This suggests that the inclusion of social capital variables significantly enhances the model’s explanatory power. Thus, both Hypothesis 1.2 and Hypothesis 2.2 are supported, indicating that the structural resources of social capital and the processes of maintaining, consolidating, strengthening, and expanding social capital have significant effects on negative health behaviors.

Models (3) and (4) present the analysis results regarding smoking frequency. Firstly, the coefficient for gender is positive and significant, indicating that females are more likely than males to have lower smoking frequencies. This finding aligns with the impact observed on alcohol consumption and is consistent with common perceptions. The effect of years of education on smoking frequency parallels its impact on alcohol consumption, as higher educational attainment significantly reduces smoking behavior. However, the influences of age, household registration status, and ethnicity are not significant. Additionally, the logarithm of household annual income significantly decreases the likelihood of individuals being in a higher smoking frequency category, while married individuals are more likely to exhibit higher smoking frequencies compared to unmarried or divorced individuals.

Finally, social capital exerts a significant influences on individual smoking behaviors. After incorporating the variables of network diversity and social dining frequency, Model (4) demonstrates a substantial improvement over Model (3) (LR chi^2^ (2) = 225.31, Prob > chi^2^ = 0.000). Both network diversity and social dining frequency significantly increase the likelihood of individuals falling into a higher smoking frequency category, with coefficients of 0.032 and 0.092, respectively, both significant at the 0.01 level. These findings further corroborate Hypotheses 1.2 and 2.2.

Based on the comprehensive analytical results, a fundamental conclusion can be drawn: social capital significantly impacts individuals’ health behaviors. Differences in the structural resources of social capital, as well as variations in the processes of maintaining, consolidating, strengthening, and broadening these resources, lead to disparities in health behaviors. Furthermore, this influence is observed across both positive and negative health behaviors.

### Propensity score matching

To investigate the effects of network differences and the frequency of social dining on health behaviors while mitigating potential biases, propensity score matching was employed (refer to Table 4 for results). The analysis revealed a consistent positive relationship between network diversity and health-promoting behaviors. Specifically, the matched estimates revealed that each unit increase in network diversity was associated with higher odds of engaging in regular exercise (OR = 1.09, 95% CI: 1.01–1.17, *p* = 0.032) and undergoing preventive health check-ups (OR = 1.16, 1.03–1.31, *p* = 0.015). These findings aligns with Berkman’s social cohesion theory, which posits that diverse social networks facilitate greater access to health information and foster shared responsibilities (59). However, the observed reduction in coefficients - such as a 33% decrease in the exercise *β* after mathching - implies that socioeconomic factors partially confound these relationships. In contrast, the frequency of dining demonstrated a dual effect: while higher frequency was linked to an increased risk of alcohol consumption (OR = 1.10, *p* = 0.013), it was unexpectedly correlated with a decreased risk of smoking (OR = 0.84, *p* = 0.019), further supporting the robustness of our findings.

**Table 4:** Effects of social capital on health behaviors (Ordered Logistic Regression).

Predictor	Outcome	Model	*β* (SE)	OR [95% CI]	*p*
Network diversity	Exercise frequency (5-level)	Pre-matched	0.12 (0.04)	1.13 [1.05, 1.21]	0.002
		Post-matched	0.08 (0.03)	1.09 [1.01, 1.17]	0.032
	Health screening (3-level)	Pre-matched	0.21 (0.05)	1.23 [1.12, 1.36]	0.001
		Post-matched	0.15 (0.06)	1.16 [1.03, 1.31]	0.015
Social dining frequency	Alcohol use (5-level)	Pre-matched	0.18 (0.05)	1.20 [1.08, 1.32]	0.001
		Post-matched	0.10 (0.04)	1.10 [1.02, 1.19]	0.013
	Smoking frequency (5-level)	Pre-matched	−0.09 (0.06)	0.91 [0.81, 1.03]	0.136
		Post-matched	−0.17 (0.07)	0.84 [0.73, 0.97]	0.019

*β*, Ordered logit coefficient; OR, exponentiated *β*; Matching: Nearest-neighbor propensity score matching (caliper = 0.2); All post-match SMDs < 0.1.

## Discussion

Health behaviors are critical determinants of individuals’ health outcomes, and exploring the social factors underlying these behaviors is significant for understanding their broader health implications. Since Wilkinson proposed social capital as a mediating factor between socioeconomic status and health (60), it has garnered considerable scholarly attention as a mechanism for explaining health outcomes. While the positive effects of social capital on health are widely recognized, it is essential to acknowledge that social capital is a multifaceted construct whose impact varies across different dimensions and contexts. Moreover, much of the existing empirical evidence is derived from developed countries, with relatively limited research focusing on populations in China. This gap in the literature raises important questions regarding which theoretical perspectives best capture the realities of developing countries like China. Therefore, further research is needed to explore the nuanced influence of social capital on health behaviors within the specific social and cultural contexts of China, in order to develop more targeted public health strategies that resonate with the unique characteristics of these populations.

Based on the JSNET 2014 data, this study demonstrates that social capital exerts a significant influence on both positive and negative health behaviors. The positive correlation between social capital and health-promoting behaviors, such as physical activity and regular health check-ups, aligns with previous research highlighting the role of social networks in encouraging healthy lifestyle choices (61). The findings indicate that individuals embedded within diverse social networks are more likely to engage in beneficial health practices due to enhanced access to resources, information, and emotional support. Conversely, the relationship between social capital and negative health behaviors, such as increased drinking and smoking frequency, reveals the dual nature of social networks. While these networks can support positive behaviors, they may also propagate unhealthy habits. This duality aligns with the findings of Christakis and Fowler, who noted that conditions such as obesity can spread through social ties, illustrating that social influences operate bidirectionally (36). This finding underscore the complexity of social capital’s role in shaping health behaviors and suggest that public health interventions must account for both the supportive and detrimental aspects of social networks.

The underlying mechanism can be explained by two distinct transmission pathways within social networks. On the one hand, based on social learning theory, individuals with high social capital tend to adopt positive health practices by observing and imitating those of their network members (e.g., group exercise, health information sharing). This form of vertical diffusion is particularly prominent in networks dominated by strong relationships (62). On the other hand, the peer influence model suggests that social capital may also contribute to negative behaviors through normative group pressures. In the Chinese context, this is exemplified by the relational ethos, which compels individuals to comform to social rituals - such as the culture of toasting at business banquets - in order to preserve relational capital, thereby passively engaging in risky behaviors such as smoking and drinking (63). This ambivalence stems from the neutral nature of social capital as a “structural vehicle” - its health effects depend on the dominant behavioral paradigms and cultural scripts within the network. For instance, strong-tie networks often promote pro-health norms in a differentiated pattern of association, whereas instrumental weak-tie networks are more influenced by face-saving expectations and reciprocal obligations, making them more susceptible to harmful behaviors (64).

For individuals in China, social capital can simultaneously contribute to unhealthy behaviors while enhancing social status and overall well-being. This study finds that the accumulation of social capital among Chinese residents leads to both beneficial and detrimental health behaviors. Chen and Bian demonstrated that social dining can undermine trust in the government through the spread of information and the pursuit of goals, which they referred to as the “side effects” of social capital. Rather than focusing solely on these negative outcomes, this study emphasizes the processes through which social capital is generated, maintained, and reinforced (57). Our analysis reveals that social capital does have certain adverse effects, which arise not only from its inherent interconnectedness but also from the distinctive social dining culture in China. This interconnectedness encompasses both positive and negative interactions, as illustrated by social dining practices.

Consequently, for theorists and policymakers at the community level, it is suggested that local social networks, such as square dance groups and homeowners’ associations, be utilized to certify “community health councilors” who possess health literacy. Additionally, group norms should be modified through the implementation of a “health point” system, where participation in group exercise can be exchanged for green passes for medical checkups. At the organizational level, the government should lead the establishment of a “health partner” responsibility system, mandating industries with high tobacco and alcohol use to appoint employees with exemplary health habits as “informal health supervisors.” This could also involve replacing alcohol with tea and offering healthy platters during high-risk situations, like business dinners. At the individual level, the development of an ecological “Social Health Portrait” app will automatically identify high-risk social circles, such as frequent attendees of drinking parties, by analyzing users’ consumption data. Furthermore, the introduction of a “Healthy Social Credit Score” system will integrate health behavior data into personal credit assessments, creating a “healthy social credit score” framework. This approach combines the vertical spread of social learning theory, the horizontal constraints of peer influence, and the relational governance characteristic of China’s unique order, achieving a significant shift from broad advocacy to targeted network governance.

Furthermore, this study has several limitations. First, there are limitations related to the data. The analysis is based on cross-sectional data, which restricts the ability to address endogeneity concerns. Due to the selectivity of social interactions, causal proofs of social capital effects face great trouble with endogeneity. This study provides preliminary evidence on the relationship between social capital and health behaviors; future research could employ more robust study designs or advanced econometric models to conduct more rigorous tests. Second, the JSNET survey was conducted solely in urban areas, meaning that the findings primarily reflect the experiences of urban populations. It remains unclear how social capital influences the health behaviors of rural populations and whether these effects differ from those observed in urban settings. More comprehensive data analysis and validation are needed in this regard. Third, the concept and measurement of social capital are quite complex. This study only examines network diversity and social dining frequency; there are many other aspects worth exploring. Future research should conduct more detailed, in-depth, and comprehensive analyses of how social capital affects various health behaviors.

## Conclusion

This study underscores the significant influence of social capital on health behaviors, revealing its dual role in promoting both positive and negative dimensions. The findings suggest that while social capital facilitates engagement in health-promoting behaviors, such as regular physical activity and preventive health check-ups, it also contributes to the persistence of negative health behaviors, including increased drinking and smoking.

This duality highlights the complex and context-dependent nature of social networks, which can provide valuable resources, information, and emotional support, while also simultaneously enabling the spread of unhealthy habits. This complexity reflects the intricate interplay between social capital and health, particularly within the unique cultural context of China.

Given these insights, there is a critical need for policymakers and researchers to leverage the beneficial aspects of social capital while curbing its potential harms. Future research should address the limitations identified, including endogeneity issues, and explore the impacts of social capital on diverse populations and health behaviors in greater depth. By doing so, a more comprehensive understanding of social capital’s role in health can be achieved, ultimately informing strategies to improve public health outcomes.

## Data Availability

The data analyzed in this study is subject to the following licenses/restrictions: data is only available to specific researchers or organizations. Requests to access these datasets should be directed to niulong@xjtu.edu.cn.
